# Supplementary material for: Collective Immunity to the Measles, Mumps, and Rubella Viruses in the Kyrgyz Population
Source: Vaccines (Basel). 2025 Feb 27;13(3):249. doi: 10.3390/vaccines13030249 (PMC11945377; doi:10.3390/vaccines13030249)
Supplement: Supplementary file 1 [file vaccines-13-00249-s001.zip › Supplement data_Table S1 edited.pdf]

Table S1. Measles seroprevalence by age group.

Age Interval, years	N	IgG+		
		n	%	95% C. I.
1–5	909	600	66.0	62.9–69*
6–11	1025	625	61.0	57.9–64*
12–17	877	532	60.7	57.3–63.9*
18–29	668	569	85.2	82.3–87.8#
30–39	686	598	87.2	84.4–89.6#
40–49	698	654	93.7	91.6–95.4#
50–59	693	652	94.1	92.1–95.7#
60–69	654	621	95.0	93–96.5#
70+	407	372	91.4	88.2–93.9#
Total:	6617	5223	78.9	77.9–79.9

Note: N — individuals, n — seropositive individuals, % — share seropositive individuals, 95% C.I. — 95% confidence interval, * — significantly lower than overall, # — significantly higher than overall.
