# Supplementary material for: Collective Immunity to the Measles, Mumps, and Rubella Viruses in the Kyrgyz Population
Source: Vaccines (Basel). 2025 Feb 27;13(3):249. doi: 10.3390/vaccines13030249 (PMC11945377; doi:10.3390/vaccines13030249)
Supplement: Supplementary file 1 [file vaccines-13-00249-s001.zip › Supplement data_Table S2 edited.pdf]

Table S2. Measles seroprevalence by region.

City/Region	N	IgG+		
		n	%	95% C. I.
Bishkek city	1132	921	81.4	79.0–83.6
Osh city	268	246	91.8	87.8–94.8#
Osh region	1410	1140	80.9	78.7–82.9
Batken region	563	389	69.1	65.1–72.9*
Jalal-Abad region	1218	912	74.9	72.3–77.3
Talas region	268	225	84.0	79.0–88.1
Issyk-Kul region	538	409	76.0	72.2–79.6
Naryn region	339	253	74.6	69.7–79.2
Chüy region	881	728	82.6	80.0–85.1#
Total:	6617	5223	78.9	77.9–79.9

Note: N — individuals, n — seropositive individuals, % — share seropositive individuals, 95% C.I. — 95% confidence interval, * — significantly lower than overall, # — significantly higher than overall.
