# Supplementary material for: Collective Immunity to the Measles, Mumps, and Rubella Viruses in the Kyrgyz Population
Source: Vaccines (Basel). 2025 Feb 27;13(3):249. doi: 10.3390/vaccines13030249 (PMC11945377; doi:10.3390/vaccines13030249)
Supplement: Supplementary file 1 [file vaccines-13-00249-s001.zip › Supplement data_Table S3 edited.pdf]

Table S3. Measles seroprevalence by activity.

Activity	N	IgG+		
		n	%	95% C. I.
Preschooler	648	444	68.5	64.8–72.1*
Schoolchild	1632	966	59.2	56.8–61.6*
Student	164	129	78.7	71.6–84.7
Medicine	1276	1196	93.7	92.3–95.0#
Science + the Arts	47	44	93.6	82.5–98.7#
Business	86	74	86	76.9–92.6
Education	198	167	84.3	78.5–89.1
Industrial + Transportation	51	44	86.3	73.7–94.3
State-Military Service	184	161	87.5	81.8–91.9#
Office	81	72	88.9	80.0–94.8
Information Technologies (IT)	66	55	83.3	72.1–91.4
Agriculture	173	148	85.5	79.4–90.4
Other	668	544	81.4	71.3–84.3
Unemployed	731	605	82.8	79.8–85.4
Retired	612	574	93.8	91.6–95.6#
Total:	6617	5223	78.9	77.9–79.9

Note: N — individuals, n — seropositive individuals, % — share seropositive individuals, 95% C.I. — 95% confidence interval, * — significantly lower than overall, # — significantly higher than overall.
