# Supplementary material for: Collective Immunity to the Measles, Mumps, and Rubella Viruses in the Kyrgyz Population
Source: Vaccines (Basel). 2025 Feb 27;13(3):249. doi: 10.3390/vaccines13030249 (PMC11945377; doi:10.3390/vaccines13030249)
Supplement: Supplementary file 1 [file vaccines-13-00249-s001.zip › Supplement data_Table S4 edited.pdf]

Table S4. Anti-measles titers by age group.

Age Interval, years	N	IgG titer range, IU/ml														
		<0.18			0.18–0.5			0.51–1.0			1.01–2.0			>2.0		
		n	%	95% C. I.	n	%	95% C. I.	n	%	95% C. I.	n	%	95% C. I.	n	%	95% C. I.
1–5	909	309	34.0	30.9–37.2#	307	33.8	30.7–37#	134	14.7	12.5–17.2	94	10.3	8.4–12.5*	65	7.2	5.6–9.0*
6–11	1025	400	39.0	36.0–42.1#	363	35.4	32.5–38.4#	113	11.0	9.2–13.1*	80	7.8	6.2–9.6*	69	6.7	5.3–8.4*
12–17	877	345	39.3	36.1–42.7#	257	29.3	26.3–32.4#	110	12.5	10.4–14.9	92	10.5	8.5–12.7*	73	8.3	6.6–10.4*
18–29	668	99	14.8	12.2–17.7*	195	29.2	25.8–32.8#	144	21.6	18.5–24.9#	139	20.8	17.8–24.1	91	13.6	11.1–16.5*
30–39	686	88	12.8	10.4–15.6*	235	34.3	30.7–37.9#	116	16.9	14.2–19.9	151	22.0	19–25.3	96	14.0	11.5–16.8
40–49	698	44	6.3	4.6–8.4*	151	21.6	18.6–24.9*	144	20.6	17.7–23.8#	196	28.1	24.8–31.6#	163	23.4	20.3–26.7#
50–59	693	41	5.9	4.3–7.9*	101	14.6	12.0–17.4*	133	19.2	16.3–22.3	217	31.3	27.9–34.9#	201	29.0	25.7–32.5#
60–69	654	33	5.0	3.5–7.0*	88	13.5	10.9–16.3*	84	12.8	10.4–15.7	194	29.7	26.2–33.3#	255	39.0	35.2–42.9#
70+	407	35	8.6	6.1–11.8*	48	11.8	8.8–15.3*	67	16.5	13.0–20.4	112	27.5	23.2–32.1#	145	35.6	31.0–40.5#
Total:	6617	1394	21.1	20.1–22.1	1745	26.4	25.3–27.5	1045	15.8	14.9–16.7	1275	19.3	18.3–20.2	1158	17.5	16.6–18.4

Note: N — individuals, n — individuals within titer range, % — share individuals within titer range, 95% C.I. — 95% confidence interval, * — significantly lower than overall, # — significantly higher than overall.
