# Supplementary material for: Collective Immunity to the Measles, Mumps, and Rubella Viruses in the Kyrgyz Population
Source: Vaccines (Basel). 2025 Feb 27;13(3):249. doi: 10.3390/vaccines13030249 (PMC11945377; doi:10.3390/vaccines13030249)
Supplement: Supplementary file 1 [file vaccines-13-00249-s001.zip › Supplement data_Table S5 edited.pdf]

Table S5. Measles history by age group.

Age Interval, years	N	SNV			SV			NSNV			NSV		
		n	%	95% C. I.	n	%	95% C. I.	n	%	95% C. I.	n	%	95% C. I.
1–5	826	6	0.7	0.3–1.6	0	0.0	0.0–0.0	162	19.6	17.0–22.5	658	79.7	76.8–82.3
6–11	921	4	0.4	0.2–1.1	5	0.5	0.2–1.3	182	19.8	17.3–22.5	730	79.3	76.5–81.8
12–17	724	6	0.8	0.4–1.8	2	0.3	0.1–1.0	181	25.0	22.0–28.3	535	73.9	70.6–77.0
18–29	575	7	1.2	0.6–2.5	6	1.0	0.5–2.3	207	36.0	32.2–40.0	355	61.7	57.7–65.6
30–39	578	11	1.9	1.1–3.4	3	0.5	0.2–1.5	218	37.7	33.9–41.7	346	59.9	55.8–63.8
40–49	581	12	2.1	1.2–3.6	8	1.4	0.7–2.7	227	39.1	35.2–43.1	334	57.5	53.4–61.4
50–59	536	10	1.9	1.0–3.4	7	1.3	0.6–2.7	267	49.8	45.6–54.0	252	47.0	42.8–51.2
60–69	510	3	0.6	0.2–1.7	12	2.4	1.4–4.1	216	42.4	38.1–46.7	279	54.7	50.4–59.0
70+	279	0	0.0	0.0–0.0	5	1.8	0.8–4.1	151	54.1	48.3– 59.9	123	44.1	38.4–50.0
Total:	5530	59	1.1	0.8–1.4	48	0.9	0.7–1.1	1811	32.7	31.5– 34.0	3612	65.3	64.1–66.6

Legend: SNV — “sick, never vaccinated”, SV — “sick, vaccinated”, NSV — “never sick, vaccinated”, NSNV — “never sick, never vaccinated”.
Note: N — individuals, n — individuals with history, 95% C.I. — 95% confidence interval.
