# Supplementary material for: Collective Immunity to the Measles, Mumps, and Rubella Viruses in the Kyrgyz Population
Source: Vaccines (Basel). 2025 Feb 27;13(3):249. doi: 10.3390/vaccines13030249 (PMC11945377; doi:10.3390/vaccines13030249)
Supplement: Supplementary file 1 [file vaccines-13-00249-s001.zip › Supplement data_Table S6 edited.pdf]

Table S6. Anti-measles titers by history.

History	N	IgG titer range, IU/ml																	
		<0.18			0.18-0.5			0.51-1.0			1.01-2.0			>2.0			0.18 ↔ >2.0		
		n	%	95% C. I.	n	%	95% C. I.	n	%	95% C. I.	n	%	95% C. I.	n	%	95% C. I.	Σ	%	95% C. I.
SNV	59	9	15.3	8.2–26.5	11	18.6	10.7–30.4	11	18.6	10.7–30.4	14	23.7	14.7–36.0	14	23.7	14.7–36.0	50	84.7	73.5–91.8
SV	48	3	6.3	2.1–16.8	13	27.1	16.6–41.0	3	6.3	2.1–16.8	9	18.8	10.2–31.9	20	41.7	28.8–55.7	45	93.8	83.2–97.9
NSNV	1811	301	16.6	15.0–18.4	399	22.0	20.2–24.0	326	18.0	16.3–19.8	425	23.5	21.6–25.5	360	19.9	18.1–21.8	1510	83.4	81.6–85.0
NSV	3612	887	24.6	23.2–26.0	1103	30.5	29.1–32.1	541	15.0	13.9–16.2	577	16.0	14.8–17.2	504	14.0	12.9–15.1	2725	75.4	74.0–76.8

Legend: SNV — “sick, never vaccinated”, SV — “sick, vaccinated”, NSV — “never sick, vaccinated”, NSNV — “never sick, never vaccinated”.
Note: N — individuals; n — individuals within titer range; % — n as percentage of N; Σ — sum of seropositive individuals; 95% C.I. — 95% confidence interval.
