# Supplementary material for: Collective Immunity to the Measles, Mumps, and Rubella Viruses in the Kyrgyz Population
Source: Vaccines (Basel). 2025 Feb 27;13(3):249. doi: 10.3390/vaccines13030249 (PMC11945377; doi:10.3390/vaccines13030249)
Supplement: Supplementary file 1 [file vaccines-13-00249-s001.zip › Supplement data_Table S7 edited.pdf]

Table S7. Rubella seroprevalence by age group.

Age Interval, years	N	IgG+		
		n	%	95% C. I.
1–5	909	827	91	88.9–92.8*
6–11	1025	972	94.8	93.3–96.1*
12–17	877	806	91.9	89.9–93.6*
18–29	668	616	92.2	89.9–94.1
30–39	686	665	96.9	95.4–98.1
40–49	698	678	97.1	95.6–98.2#
50–59	693	663	95.7	93.9–97.1
60–69	654	624	95.4	93.5–96.9
70+	407	385	94.6	91.9–96.6
Total:	6617	6236	94.2	93.7–94.8

Note: N — individuals, n — seropositive individuals, % — share seropositive individuals, 95% C.I. — 95% confidence interval, * — significantly lower than overall, # — significantly higher than overall.
