# Supplementary material for: Collective Immunity to the Measles, Mumps, and Rubella Viruses in the Kyrgyz Population
Source: Vaccines (Basel). 2025 Feb 27;13(3):249. doi: 10.3390/vaccines13030249 (PMC11945377; doi:10.3390/vaccines13030249)
Supplement: Supplementary file 1 [file vaccines-13-00249-s001.zip › Supplement data_Table S8 edited.pdf]

Table S8. Rubella seroprevalence by region.

City/Region	N	IgG+		
		n	%	95% C. I.
Bishkek city	1132	1072	94.7	93.2–95.9
Osh city	268	256	95.5	92.3–97.7
Osh region	1410	1335	94.7	93.4–95.8
Batken region	563	517	91.8	89.3–94.0*
Jalal-Abad region	1218	1152	94.6	93.2–95.8
Talas region	268	253	94.4	90.9–96.8
Issyk-Kul region	538	514	95.5	93.4–97.1
Naryn region	339	309	91.2	87.6–94*
Chüy region	881	828	94	92.2–95.5
Total:	6617	6236	94.2	93.7–94.8

Note: N — individuals, n — seropositive individuals, % — share seropositive individuals, 95% C.I. — 95% confidence interval, * — significantly lower than overall.
