# Supplementary material for: Collective Immunity to the Measles, Mumps, and Rubella Viruses in the Kyrgyz Population
Source: Vaccines (Basel). 2025 Feb 27;13(3):249. doi: 10.3390/vaccines13030249 (PMC11945377; doi:10.3390/vaccines13030249)
Supplement: Supplementary file 1 [file vaccines-13-00249-s001.zip › Supplement data_Table S9 edited.pdf]

Table S9. Rubella seroprevalence by activity.

Activity	N	IgG+		
		n	%	95% C. I.
Preschooler	648	598	92.3	90–94.2
Schoolchild	1632	1521	93.2	91.9–94.4
Student	164	155	94.5	89.8–97.5
Healthcare	1276	1233	96.6	95.5–97.6#
Science + the Arts	47	45	95.7	85.5–99.5
Business	86	81	94.2	87–98.1
Education	198	186	93.9	89.6–99.6
Industrial + Transportation	51	48	94.1	83.3–98.8
State-Military Service	184	174	94.6	90.2–97.4
Office	81	78	96.3	89.6–99.2
Information Technologies (IT)	66	63	95.5	87.3–99.1
Agriculture	173	163	94.2	89.6–97.2
Unemployed	731	688	94.1	92.2–95.7
Other	668	621	93.0	90.7–94.8
Retiree	612	582	95.1	93.1–96.7
Total:	6617	6236	94.2	93.7–94.4

Note: N — individuals, n — seropositive individuals, % — share seropositive individuals, 95% C.I. — 95% confidence interval, # — significantly higher than overall.
