# Supplementary material for: Collective Immunity to the Measles, Mumps, and Rubella Viruses in the Kyrgyz Population
Source: Vaccines (Basel). 2025 Feb 27;13(3):249. doi: 10.3390/vaccines13030249 (PMC11945377; doi:10.3390/vaccines13030249)
Supplement: Supplementary file 1 [file vaccines-13-00249-s001.zip › Supplement data_Table S10 edited.pdf]

Table S10. Anti-rubella titers by age group.

Age Interval, years	N	IgG titer range, IU/ml														
		<10			10.1–25.0			25.1–100			100.1–200			>200		
		n	%	95% C. I.	n	%	95% C. I.	n	%	95% C. I.	n	%	95% C. I.	n	%	95% C. I.
1–5	909	82	9	7.2–11.1	46	5.1	3.7–6.7*	317	34.9	31.8–38.1*	248	27.3	24.4–30.3#	216	23.8	21.0–26.7#
6–11	1025	53	5.2	3.9–6.7	127	12.4	10.4–14.6	546	53.3	50.2–56.4#	198	19.3	16.9–21.9	101	9.9	8.1–11.8*
12–17	877	71	8.1	6.4–10.1	175	20	17.4–22.8#	483	55.1	51.7–58.4#	99	11.3	9.3–13.6*	49	5.6	4.2–7.3*
18–29	668	52	7.8	5.9–10.1	70	10.5	8.3–13.1	370	55.4	51.5–59.2#	106	15.9	13.2–18.9	70	10.5	8.3–13.1*
30–39	686	21	3.1	1.9–4.6*	62	9	7.0–11.4	293	42.7	39.0–46.5	151	22	19.0–25.3	159	23.2	20.1–26.5#
40–49	698	20	2.9	1.8–4.4*	57	8.2	6.2–10.5	312	44.7	41.0–48.5	156	22.3	19.3–25.6	153	21.9	18.9–25.2#
50–59	693	30	4.3	2.9–6.1	58	8.4	6.4–10.7	321	46.3	42.6–50.1	140	20.2	17.3–23.4	144	20.8	17.8–24.0
60–69	654	30	4.6	3.1–6.5	61	9.3	7.2–11.8	290	44.3	40.5–48.2	129	19.7	16.7–23.0	144	22	18.9–25.4
70+	407	22	5.4	3.4–8.1	34	8.4	5.9–11.5	202	49.6	44.7–54.6	72	17.7	14.1–21.8	77	18.9	15.2–23.1
Total:	6617	381	5.8	5.2–6.4	690	10.4	9.7–11.2	3134	47.4	46.2–48.6	1299	19.6	18.7–20.6	1113	16.8	15.9–17.7

Note: N — individuals, n — individuals within titer range, % — share individuals within titer range, 95% C.I. — 95% confidence interval, * — significantly lower than overall, # — significantly higher than overall.
