# Supplementary material for: Collective Immunity to the Measles, Mumps, and Rubella Viruses in the Kyrgyz Population
Source: Vaccines (Basel). 2025 Feb 27;13(3):249. doi: 10.3390/vaccines13030249 (PMC11945377; doi:10.3390/vaccines13030249)
Supplement: Supplementary file 1 [file vaccines-13-00249-s001.zip › Supplement data_Table S11 edited.pdf]

Table S11. Rubella history by age group.

Age Interval, years	N	SNV			SV			NSNV			NSV		
		n	%	95% C. I.	n	%	95% C. I.	n	%	95% C. I.	n	%	95% C. I.
1–5	828	0	0	0.0–0.0	0	0	0.0–0.0	256	30.9	27.9–34.1*	572	69.1	65.9–72.1#
6–11	922	2	0.2	0.1–0.8	3	0.3	0.1–1.0	298	32.3	29.4–35.4*	619	67.1	64.0–70.1#
12–17	720	3	0.4	0.1–1.2	4	0.6	0.2–1.4	245	34	30.7–37.6*	468	65	61.4–68.4#
18–29	569	5	0.9	0.4–2.0	2	0.4	0.1–1.3	262	46	42.0–50.2	300	52.7	48.6–56.8
30–39	577	6	1.0	0.5–2.2	4	0.7	0.3–1.8	244	42.3	38.3–46.4	323	56	51.9–60.0
40–49	593	6	1.0	0.5–2.2	2	0.3	0.1–1.2	284	47.9	43.9–51.9	301	50.8	46.7–54.8*
50–59	538	2	0.4	0.1–1.3	4	0.7	0.3–1.9	315	58.6	54.3–62.6#	217	40.3	36.3–44.5*
60–69	519	3	0.6	0.2–1.7	2	0.4	0.1–1.4	259	49.9	45.6–54.2	255	49.1	44.9–53.4*
70+	278	0	0.0	0.0–0.0	0	0	0.0–0.0	153	55	49.2–60.8	125	45	39.2–50.8*
Total:	5544	27	0.5	0.3–0.7	21	0.4	0.2–0.6	2316	41.8	40.5–43.1	3180	57.4	56.1–58.7

Legend: SNV — “sick, never vaccinated”, SV — “sick, vaccinated”, NSV — “never sick, vaccinated”, NSNV — “never sick, never vaccinated”.
Note: N — individuals, n — individuals with history, 95% C.I. — 95% confidence interval, * — significantly lower than overall, # — significantly higher than overall.
