# Supplementary material for: Collective Immunity to the Measles, Mumps, and Rubella Viruses in the Kyrgyz Population
Source: Vaccines (Basel). 2025 Feb 27;13(3):249. doi: 10.3390/vaccines13030249 (PMC11945377; doi:10.3390/vaccines13030249)
Supplement: Supplementary file 1 [file vaccines-13-00249-s001.zip › Supplement data_Table S12 edited.pdf]

Table S12. Anti-rubella titers by history.

History	N	IgG titer range, IU/ml																	
		<10			10–25			25.1–100			100.1–200			>200			10 ↔ 200		
		n	%	95% C. I.	n	%	95% C. I.	n	%	95% C. I.	n	%	95% C. I.	n	%	95% C. I.	Σ	%	95% C. I.
SNV	27	3	11.1	3.9–28.1	5	18.5	8.2–36.7	12	44.4	27.6–62.7	5	18.5	8.2–36.7	2	7.4	2.1–23.4	24	88.9	71.9–96.1
SV	21	1	4.8	0.8–22.7	1	4.8	0.8–22.7	9	42.9	24.5–63.5	4	19.0	7.7–40.0	6	28.6	13.8–50.0	20	95.2	77.3–99.2
NSNV	2316	141	6.1	5.2–7.1	235	10.1	9.0–11.4	1070	46.2	44.2–48.2	457	19.7	18.2–21.4	413	17.8	16.3–19.4	2175	93.9	92.9–94.8
NSV	3180	159	5.0	4.3–5.8	339	10.7	9.6–11.8	1515	47.6	45.9–49.4	655	20.6	19.2–22.0	512	16.1	14.9–17.4	3021	95.0	94.2–95.7

Legend: SNV — “sick, never vaccinated”, SV — “sick, vaccinated”, NSV — “never sick, vaccinated”, NSNV — “never sick, never vaccinated”.
Note: N — individuals, n — individuals within titer range, % — n as percentage of N, Σ — sum of seropositive individuals, 95% C.I. — 95% confidence interval
