# Supplementary material for: Collective Immunity to the Measles, Mumps, and Rubella Viruses in the Kyrgyz Population
Source: Vaccines (Basel). 2025 Feb 27;13(3):249. doi: 10.3390/vaccines13030249 (PMC11945377; doi:10.3390/vaccines13030249)
Supplement: Supplementary file 1 [file vaccines-13-00249-s001.zip › Supplement data_Table S13 edited.pdf]

Table S13. Mumps seroprevalence by age group.

Age Interval, years	N	IgG+		
		n	%	95% C. I.
1–5	909	695	76.5	73.6–79.2
6–11	1025	841	82	79.6–84.4#
12–17	877	576	65.7	62.4–68.8*
18–29	668	410	61.4	57.6–65.1*
30–39	686	511	74.5	71.1–77.7
40–49	698	555	79.5	76.3–82.5
50–59	693	575	83	80–85.7#
60–69	654	558	85.3	82.4–88#
70+	407	333	81.8	77.7–85.5#
Total:	6617	5054	76.4	75.3–77.4

Note: N — individuals, n — seropositive individuals, % — share seropositive individuals, 95% C.I. — 95% confidence interval, * — significantly lower than overall, # — significantly higher than overall
