# Supplementary material for: Collective Immunity to the Measles, Mumps, and Rubella Viruses in the Kyrgyz Population
Source: Vaccines (Basel). 2025 Feb 27;13(3):249. doi: 10.3390/vaccines13030249 (PMC11945377; doi:10.3390/vaccines13030249)
Supplement: Supplementary file 1 [file vaccines-13-00249-s001.zip › Supplement data_Table S14 edited.pdf]

Table S14. Mumps seroprevalence by region.

City/Region	N	IgG+		
		n	%	95% C. I.
Bishkek city	1132	873	77.1	74.6–79.5
Osh city	268	202	75.4	69.8–80.4
Osh region	1410	1124	79.7	77.5–81.8
Batken region	563	434	77.1	73.4–80.5
Jalal-Abad region	1218	963	79.1	76.7–81.3
Talas region	268	197	73.5	67.8–78.7
Issyk-Kul region	538	362	67.3	63.1–71.2
Naryn region	339	245	72.3	67.2–77.0
Chüy region	881	654	74.2	71.2–77.1
Total:	6617	5054	76.4	75.3–77.4

Note: N — individuals, n — seropositive individuals, % — share seropositive individuals, 95% C.I. — 95% confidence interval.
