# Supplementary material for: Collective Immunity to the Measles, Mumps, and Rubella Viruses in the Kyrgyz Population
Source: Vaccines (Basel). 2025 Feb 27;13(3):249. doi: 10.3390/vaccines13030249 (PMC11945377; doi:10.3390/vaccines13030249)
Supplement: Supplementary file 1 [file vaccines-13-00249-s001.zip › Supplement data_Table S15 edited.pdf]

Table S15. Mumps seroprevalence by activity.

Activity	N	IgG+		
		n	%	95% C. I.
Preschooler	648	509	78.5	75.2–81.7
Schoolchild	1632	1241	76	73.9–78.1
Student	164	83	50.6	42.7–58.5*
Healthcare	1276	1009	79.1	76.7–81.3
Science + the Arts	47	34	78.7	64.3–89.3
Business	86	71	82.6	72.9–89.9
Education	198	150	75.8	69.2–81.6
Industrial + Transportation	51	39	76.5	62.5–87.2
State-Military Service	184	143	77.7	71.0–83.5
Office	81	54	66.7	55.3–76.8
Information Technologies (IT)	66	55	83.3	72.1–91.4
Agriculture	173	131	75.7	68.6–81.9
Other	668	486	72.7	69.2–76.1
Unemployed	731	525	71.8	68.4–75.1
Retired	612	524	85.6	82.6–88.3#
Total:	6617	5054	76.4	75.3–77.4

Note: N — individuals, n — seropositive individuals, % — share seropositive individuals, 95% C.I. — 95% confidence interval, * — significantly lower than overall, # — significantly higher than overall.
