# Supplementary material for: Collective Immunity to the Measles, Mumps, and Rubella Viruses in the Kyrgyz Population
Source: Vaccines (Basel). 2025 Feb 27;13(3):249. doi: 10.3390/vaccines13030249 (PMC11945377; doi:10.3390/vaccines13030249)
Supplement: Supplementary file 1 [file vaccines-13-00249-s001.zip › Supplement data_Table S16 edited.pdf]

Table S16. Mumps history by age group.

Age Interval, years	N	SNV			SV			NSNV			NSV		
		n	%	95% C. I.	n	%	95% C. I.	n	%	95% C. I.	n	%	95% C. I.
1–5	818	3	0.4	0.1–1.1	0	0	0.0–0.0	260	31.8	28.7–35.1	555	67.8	64.6–71.0
6–11	915	4	0.4	0.2–1.1	5	0.5	0.2–1.3	319	34.9	31.8–38.0	587	64.2	61.0–67.2
7–12	711	3	0.4	0.1–1.2	4	0.6	0.2–1.4	255	35.9	32.4–39.5	449	63.2	59.5–66.6
18–29	562	6	1.1	0.5–2.3	6	1.1	0.5–2.3	266	47.3	43.2–51.5	284	50.5	46.4–54.6
30–39	575	7	1.2	0.6–2.5	6	1	0.5–2.3	259	45	41.0–49.1	303	52.7	48.6–56.7
40–49	596	6	1	0.5–2.2	11	1.8	1.0–3.3	300	50.3	46.3–54.3	279	46.8	42.8–50.8
50–59	541	7	1.3	0.6–2.6	3	0.6	0.2–1.6	332	61.4	57.2–65.4	199	36.8	32.8–40.9
60–69	526	4	0.8	0.3–1.9	5	1	0.4–2.2	278	52.9	48.6–57.1	239	45.4	41.2–49.7
70+	273	1	0.4	0.1–2.0	3	1.1	0.4–3.2	155	56.8	50.8–62.5	114	41.8	36.1–47.7
Total:	5517	41	0.7	0.5–1.0	43	0.8	0.6–1.0	2424	43.9	42.6–45.3	3009	54.5	53.2–55.9

Legend: SNV — “sick, never vaccinated”, SV — “sick, vaccinated”, NSV — “never sick, vaccinated”, NSNV — “never sick, never vaccinated”.
Note: N — individuals, n — individuals with history, 95% C.I. — 95% confidence interval.
