# Supplementary material for: Collective Immunity to the Measles, Mumps, and Rubella Viruses in the Kyrgyz Population
Source: Vaccines (Basel). 2025 Feb 27;13(3):249. doi: 10.3390/vaccines13030249 (PMC11945377; doi:10.3390/vaccines13030249)
Supplement: Supplementary file 1 [file vaccines-13-00249-s001.zip › Supplement data_Table S17 edited.pdf]

Table S17. Mumps seroprevalence by history.

Age Interval, years	SNV				SV				NSNV				NSV			
	N	n	%	95% C. I.	N	n	%	95% C. I.	N	n	%	95% C. I.	N	n	%	95% C. I.
1–5	3	3	100	43.8–100.0	0	0	0	0.0–0.0	260	194	74.6	69.0–79.5	555	434	78.2	74.6–81.4
6–11	4	4	100	51.0–100.0	5	5	100	56.6–100.0	319	258	80.9	76.2–84.8	587	492	83.8	80.6–86.6
12–17	3	1	33.3	6.1–79.2	4	4	100	51.0–100.0	255	163	63.9	57.9–69.6	449	305	67.9	63.5–72.1
18–29	6	4	66.7	30.0–90.3	6	5	83.3	43.6–97.0	266	169	63.5	57.6–69.1	284	157	55.3	49.5–61.0
30–39	7	6	85.7	48.7–97.4	6	5	83.3	43.6–97.0	259	192	74.1	68.5–79.1	303	223	73.6	68.4–78.2
40–49	6	5	83.3	43.6–97.0	11	8	72.7	43.4–90.3	300	245	81.7	76.9–85.6	279	219	78.5	73.3–82.9
50–59	7	7	100	64.6–100.0	3	3	100	43.8–100.0	332	276	83.1	78.7–86.8	199	168	84.4	78.7–88.8
60–69	4	4	100	51.0–100.0	5	4	80	37.6–96.4	278	233	83.8	79.0–87.7	239	202	84.5	79.4–88.6
70+	1	1	100	20.7–100.0	3	3	100	43.8–100.0	155	124	80	73.0–85.5	114	97	85.1	77.4–90.5
Total:	41	35	85.4	71.6–93.1	43	37	86	72.7–93.4	2424	1854	76.5	74.8–78.1	3009	2297	76.3	74.8–77.8

Legend: SNV — “sick, never vaccinated”, SV — “sick, vaccinated”, NSV — “never sick, vaccinated”, NSNV — “never sick, never vaccinated”.
Notes: N — individuals with the specified history, n — number of seropositive individuals in the age group, 95% C.I. — 95% confidence interval.
