# Supplementary material for: Collective Immunity to the Measles, Mumps, and Rubella Viruses in the Kyrgyz Population
Source: Vaccines (Basel). 2025 Feb 27;13(3):249. doi: 10.3390/vaccines13030249 (PMC11945377; doi:10.3390/vaccines13030249)
Supplement: Supplementary file 1 [file vaccines-13-00249-s001.zip › Supplementary materials_List_of_tables edited.pdf]

Supplementary Materials

List of Tables

Table S1. Measles seroprevalence by age group.
Table S2. Measles seroprevalence by region.
Table S3. Measles seroprevalence by activity.
Table S4. Anti-measles titers by age group.
Table S5. Measles history by age group.
Table S6. Anti-measles titers by history.

Table S7. Rubella seroprevalence by age group.
Table S8. Rubella seroprevalence by region.
Table 9. Rubella seroprevalence by activity.
Table S10. Anti-rubella titers by age group.
Table S11. Rubella history by age group.
Table S12. Anti-rubella titers by history.

Table S13. Mumps seroprevalence by age group.
Table S14. Mumps seroprevalence by region.
Table S15. Mumps seroprevalence by activity.
Table S16. Mumps history by age group.
Table S17. Mumps seroprevalence by history.
